# Supplementary material for: Dynamic neural representations of memory and space during human ambulatory navigation
Source: Nat Commun. 2023 Oct 20;14:6643. doi: 10.1038/s41467-023-42231-4 (PMC10589239; doi:10.1038/s41467-023-42231-4)
Supplement: Supplementary file 1 — Supplementary Information [file 41467_2023_42231_MOESM1_ESM.pdf]

Supplementary Information

Dynamic neural representations of memory and space during human ambulatory navigation

Maoz et al

Supplementary Figures

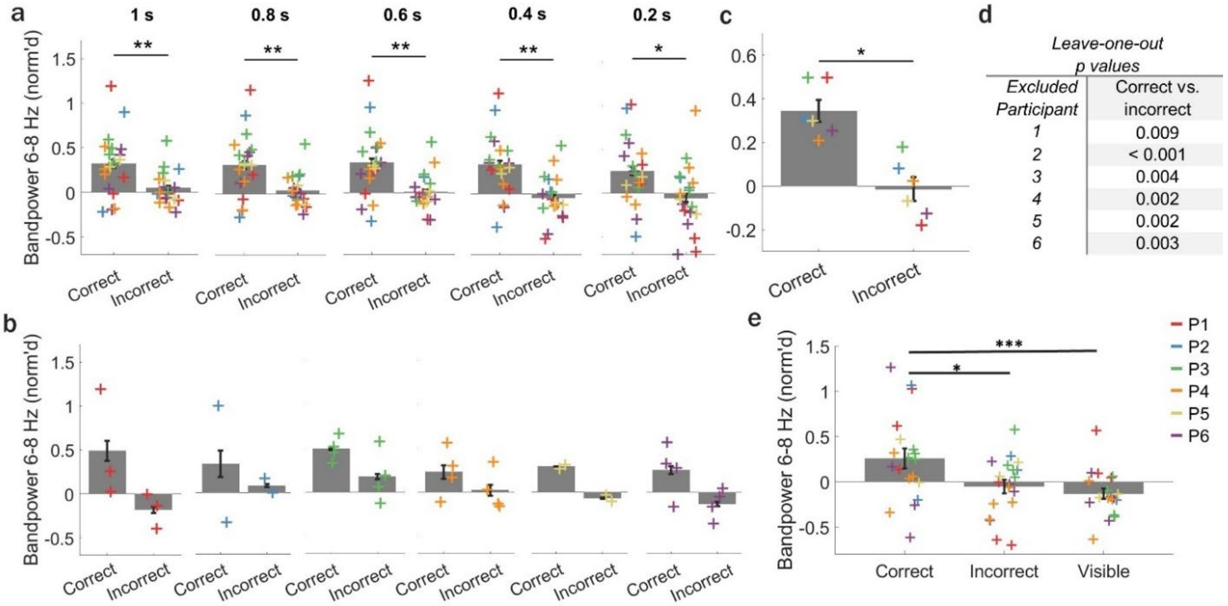

Supplementary Fig. 1: Supplementary analyses for successful memory modulation of theta. (a-c) MTL theta band power in the 0.5 s prior to arrival at the recalled target position (button press) for correct and incorrect trials **(a)** across various time windows within 1 s (0.2 – 1 s, $n_{\text{channels}} = 19$), **(b)** illustrated across channels in individual participants ($n_{P1} = 3$, $n_{P2} = 2$, $n_{P3} = 4$, $n_{P4} = 4$, $n_{P5} = 2$, $n_{P6} = 4$), and **(c)** when averaging over individual channels per participant ($n_{\text{participants}} = 6$). **(d)** Correct vs. incorrect increases in MTL theta band power remained significant when using a leave-one-out approach where each of the 6 participants were excluded one at a time. **(e)** MTL theta band power significantly increased during correct relative to incorrect retrieval trials and visible feedback periods for non-boundary positions, corrected using false discovery rate [FDR] ($n_{\text{channels}} = 19$). Crosses (+) represent the mean norm'd (normalized) band power across all trials for **(a,b,e)** individual channels and **(c)** across channels in a participant where separate colors correspond to individual participants. * = $p < 0.05$, ** = $p < 0.01$, *** = $p < 0.001$. Source data are provided as a Source Data file.

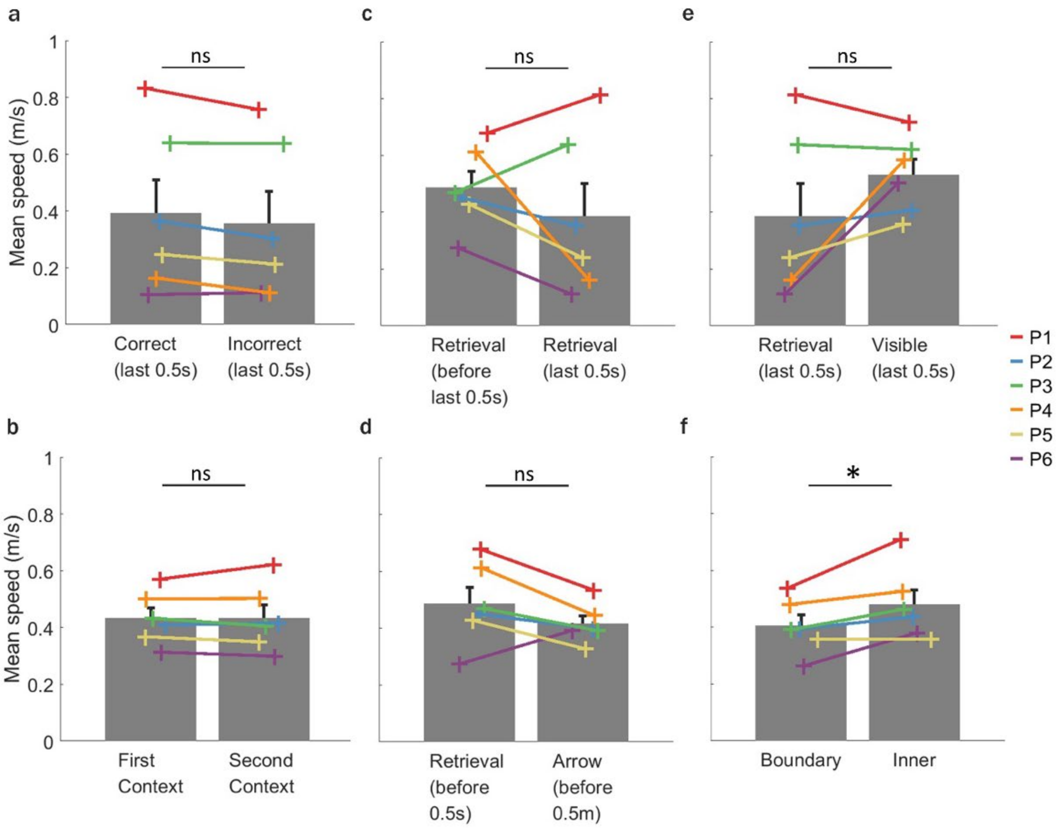

Supplementary Fig. 2: Movement speed during different task conditions. Mean speed (± standard error of the mean [s.e.m.]) across participants (crosses (+), $n_{participants} = 6$) compared between task conditions for **(a)** correct versus incorrect retrieval trials during the 0.5 s prior to arrival at the recalled target position (button press), **(b)** first versus second context, **(c)** before versus during the 0.5 s prior to button press, **(d)** retrieval (excluding 0.5 s prior to button press) versus arrow (excluding 0.5 m preceding arrival at arrow) trials, **(e)** retrieval (excluding 0.5 s prior to button press) versus visible feedback (excluding 0.5 s preceding arrival at visible halo position), and **(f)** positions in the boundary (< 1.2 m from walls) versus inner area (> 1.2 m from walls) of the room. ns = $p > 0.05$, * = $p < 0.05$. Source data are provided as a Source Data file.

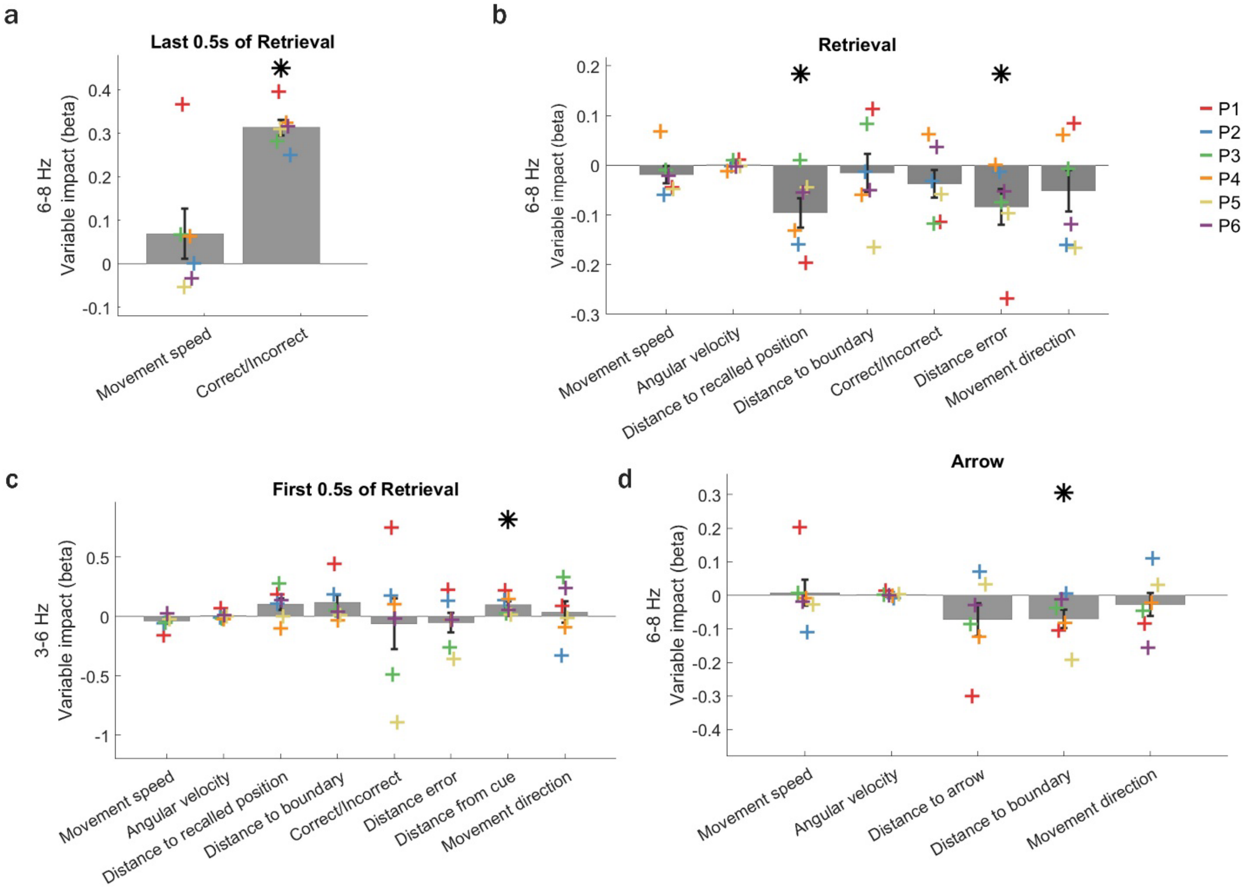

Supplementary Fig. 3: Simultaneous impact of multiple variables on theta power. Linear mixed-effects models were calculated to predict each participant's normalized theta band power timeseries by a range of predictor variables that were fixed effects with samples blocked according to channel identity. Asterisks denote a significant impact of a variable's beta weight on theta power, * = $p < 0.05$ for $n_{\text{participants}} = 6$. Error bars show the s.e.m. of the beta weights across participants. Models were implemented for **(a)** the last 0.5 s of retrieval (when no halos were visible), **(b)** the entire retrieval period (when no halos were visible), **(c)** the first 0.5 s of retrieval (after cue onset), and **(d)** the arrow search period. Movement speed, angular velocity, distance to recalled position (distance in meters to the button press location), distance from cue (distance in meters from the retrieval cue), distance to boundary (distance in meters to the nearest wall), distance to arrow (distance in meters to the target arrow), and distance error (distance in meters between the recalled position and target location) were included as continuous variables. A significant negative impact of a distance variable (i.e., distance to recalled position, distance from cue, distance error, distance to arrow, and distance to boundary) reflects increased theta band power for shorter distances. Whether a trial was correct or incorrect (correct/incorrect) was a binary variable and movement direction was a categorical variable comprised of 12 possible binned movement directions (with the mean beta coefficient over all 12 directional bins depicted). A significant positive impact of the correct/incorrect variable signifies higher theta band power values observed for correct compared to incorrect trials. Crosses (+) represent the variable impact (beta) colored according to participant. Source data are provided as a Source Data file.

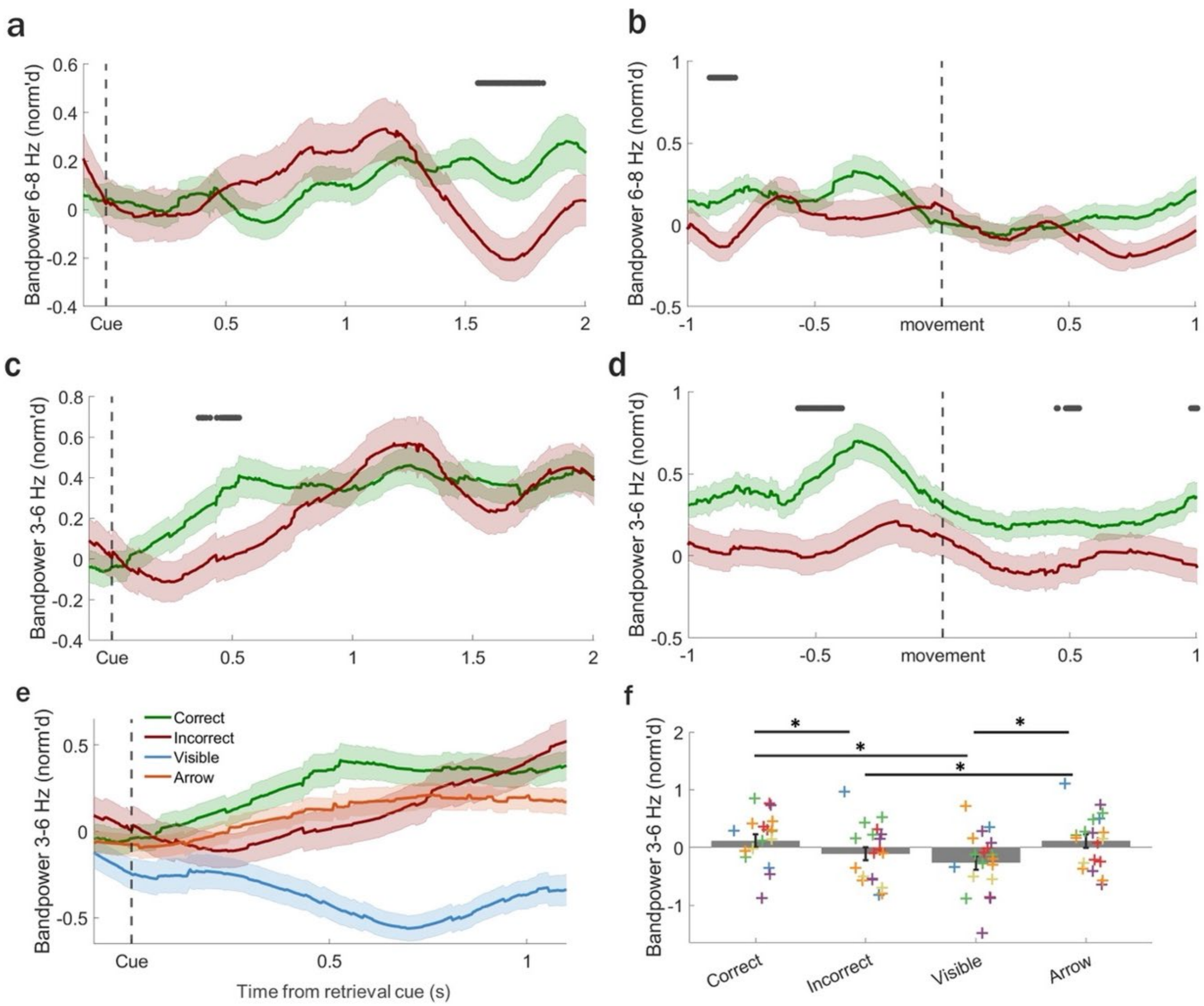

Supplementary Fig. 4: Successful memory modulation of theta band power after cue and movement onset. Mean (± s.e.m.) norm'd (normalized) theta band power across MTL channels (n_{channels} = 19) **(a,c)** after cue presentation and **(b,d)** around movement onset (last movement onset prior to button press in each retrieval trial) in **(a-b)** 6-8 Hz and **(c-d)** 3-6 Hz band power. Note, halos were not visible during correct (green) or incorrect (red) retrieval trials. Gray bar indicates timepoints where $p < 0.05$ (one-sided permutation test at each time point, representing 4 ms steps at 250 Hz sampling rate). **(e)** Mean (± s.e.m.) norm'd theta band power across MTL channels (n_{channels} = 19). Vertical gray dotted line (time = 0) indicates trial onset when the instructional cue was presented to navigate to the target halo during retrieval trials, visible halo (visible, blue) during feedback, or arrow (orange) during arrow trials. **(f)** Mean (± s.e.m.) norm'd theta band power across MTL channels (n_{channels} = 19) during 0.25-0.5 s after cue for correct/incorrect trials, visible feedback periods, and arrow trials. MTL theta band power significantly increased during this period after cue for correct compared to incorrect and visible (feedback) trials. Crosses (+) represent the mean norm'd band power across all trials for an individual channel with each color corresponding to channels from a single participant. * = $p < 0.05$, ** = $p < 0.01$, *** = $p < 0.0001$, FDR corrected. Source data are provided as a Source Data file.

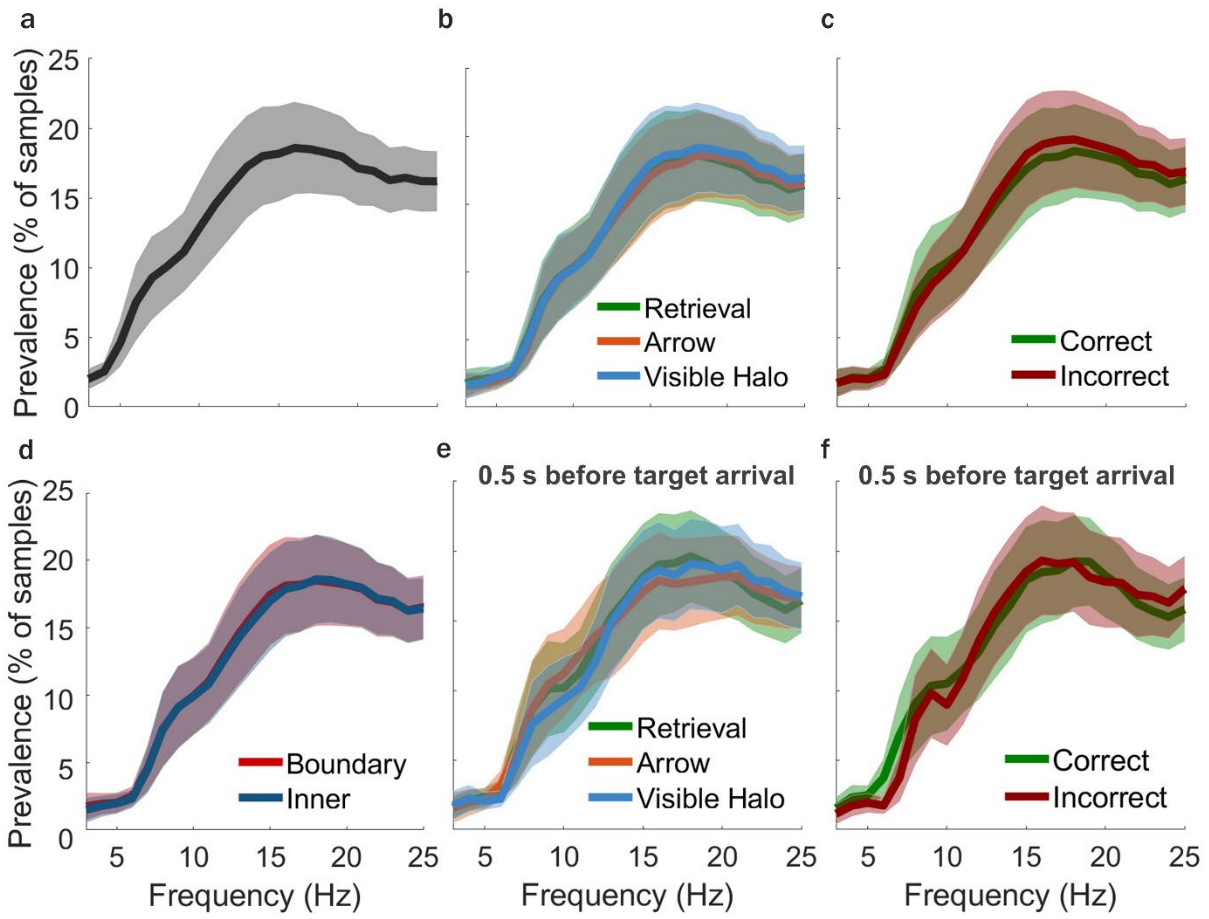

Supplementary Fig. 5: Oscillatory prevalence across task conditions. Mean (± standard error of the mean [s.e.m.]) oscillatory prevalence across frequencies (3 – 25 Hz) and MTL channels (n_{channels} = 19) during **(a)** the entire task**, (b)** retrieval, arrow, and visible halo trials, **(c)** correct and incorrect retrieval trials, **(d)** boundary (< 1.2 m from room walls) and inner (> 1.2 m from room walls) room positions, **(e)** retrieval, arrow, and visible halo trials (0.5 s before target arrival), and **(f)** correct and incorrect retrieval trials (0.5 s before arrival at the target). No significant differences were found in oscillatory prevalence across frequencies (3 – 25 Hz) for any conditions shown **(b-f).** p > 0.05 for all frequencies, FDR corrected. Source data are provided as a Source Data file.

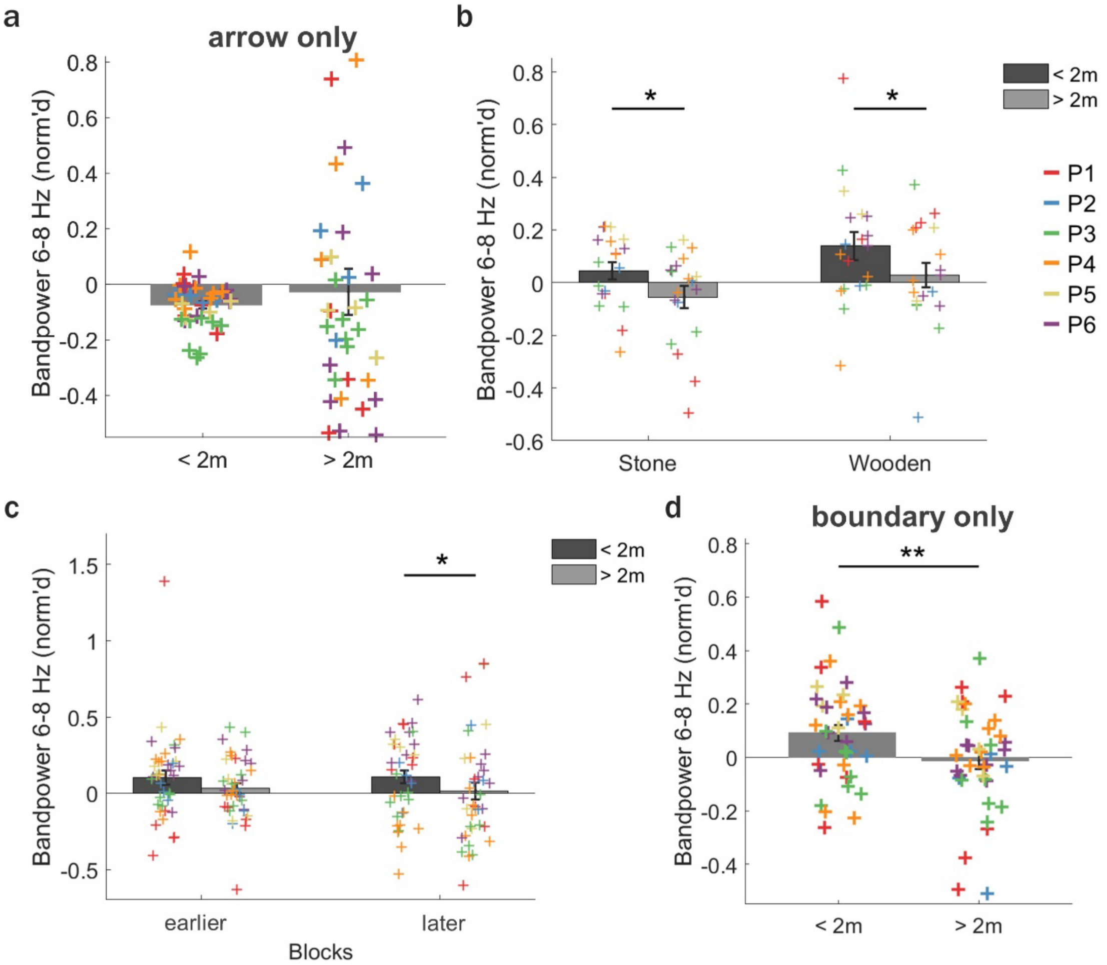

e

Threshold	*1.25 m*		*1.5 m*		*2 m*	
	% of Close	% of Far	% of Close	% of Far	% of Close	% of Far
Boundary	51.4%	91.6%	54.3%	96.8%	62.9%	99.7%
Inner	48.6%	8.4%	45.7%	3.2%	37.1%	0.3%
Total Room	48.1%	51.9%	57.7%	42.3%	74.5%	25.5%

Supplementary Fig. 6: Close vs. far control analyses. (a-d) Mean normalized (norm'd) theta band power in positions close to (< 2 m) versus far away (> 2 m) from non-target halo centers **(a)** was not significantly different when computed during arrow trials, **(b)** was significantly increased within each context, **(c)** was significantly increased during later blocks but not in earlier blocks (one-sided permutation test) and **(d)** was significantly increased when analyzed only in boundary positions. **(e)** Percentages of “close” and “far” regions that fall into boundary and inner positions for a 1.25 m, 1.5 m, and 2 m threshold. Total percentage of room area that falls into “close” and “far” regions is also listed. Crosses (+) represent the mean norm’d band power for each channel (n_{channels} = 19) during retrieval trials in each context, excluding the last 0.5 s prior to arrival at the recalled target position (button press) and are colored according to participant. * = $p < 0.05$, ** = $p < 0.01$. Source data are provided as a Source Data file.

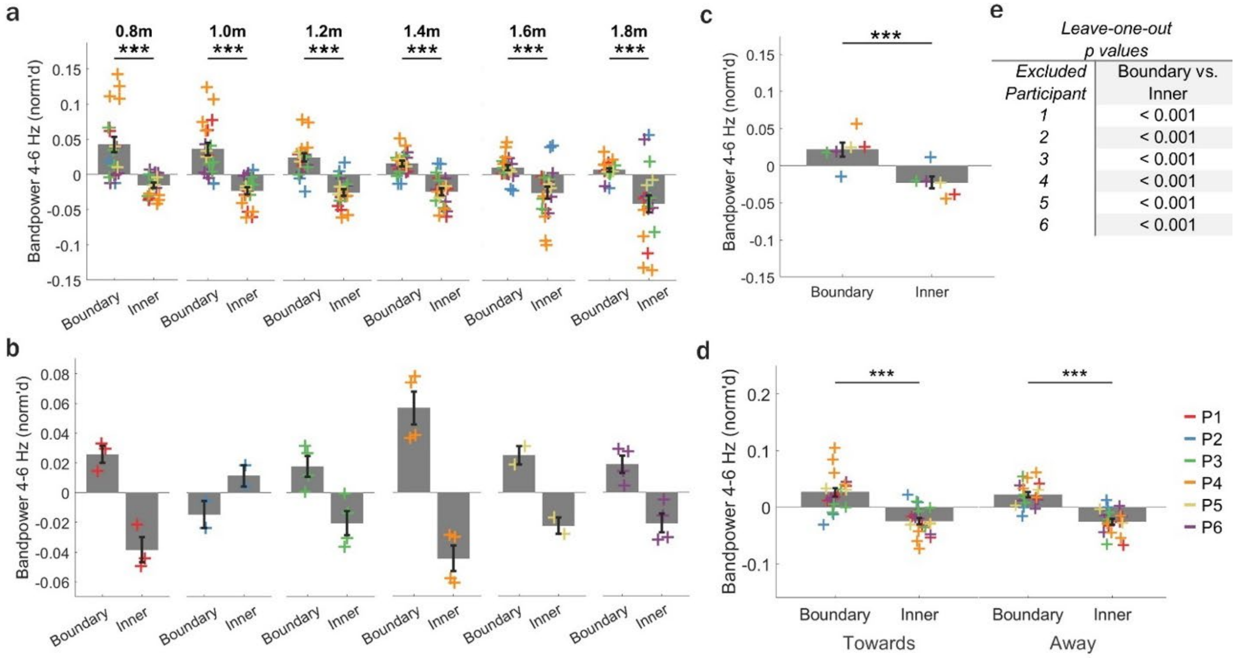

Supplementary Fig. 7: Boundary versus inner distance thresholds and control analyses. (a-d) Mean ± s.e.m. normalized (norm'd) theta band power (4-6 Hz) during arrow trials, after excluding the 0.5 m prior to participant arrival to a visible arrow on each trial **(a)** across MTL channels (n_{channels} = 19) for boundary and inner positions using varying threshold definitions for boundary versus inner cutoff ranging between 0.8 - 1.8 m, **(b)** in MTL channels for each participant for boundary and inner positions using a 1.2 m threshold (n_{P1} = 3, n_{P2} = 2, n_{P3} = 4, n_{P4} = 4, n_{P5} = 2, n_{P6} = 4), **(c)** when averaging across each participant's individual channels ($n_{\text{participants}}$ = 6), and **(d)** for instances when participants are moving towards (left) or away (right) from boundaries (n_{channels} = 19). Crosses (+) represent mean power averaged over MTL channels for each participant. *** = p < 0.001, one-sided pairwise permutation test. **(e)** Leave-one-out approach with analysis run after excluding one participant each time and associated statistic shown. Source data are provided as a Source Data file.

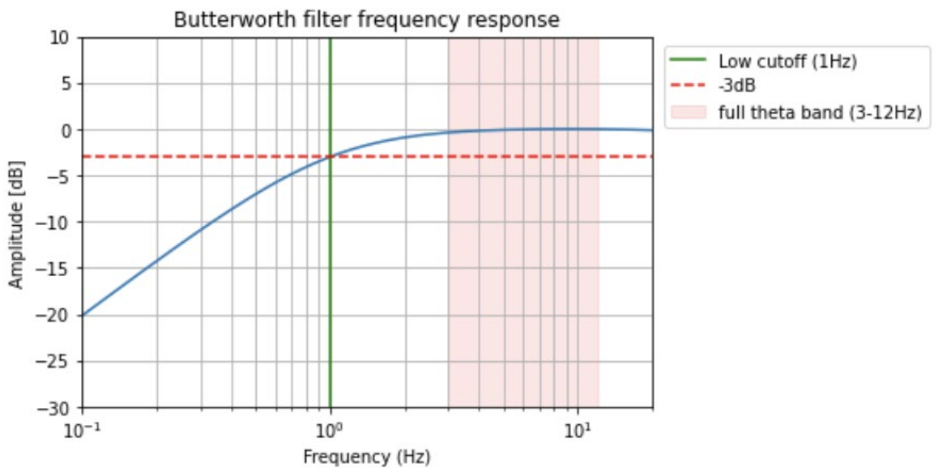

Supplementary Fig. 8: Hardware filter response. Illustration of filter response profile of RNS System hardware filter settings using a 1st order Butterworth band pass filter in 1-90 Hz range with 3 dB attenuation. Broad theta band power range (3-12 Hz, shaded in red) does not undergo amplitude attenuation (blue line shows amplitude attenuation of 0) and is above the low cutoff at 1 Hz (green line). Source data are provided as a Source Data file.

Participant		P1	P2	P3	P4	P5	P6
**Age**		50	38	43	54	33	42
**Sex**		female	male	male	female	female	female
**Total retrieval blocks (stone + wooden contexts):**		6	12	18	17	20	8
**# of trials in retrieval block #1 (Stone)**		30	15	18	18	15	69
**# of trials in retrieval block #1 (Wooden)**		21	30	27	18	21	60
**Recording duration (minutes):**		32	143	114	103	155	135
**Electrode 1**	*Hemisphere*	left	left	left	left	left	left
	Contact Spacing (mm)	3.5	10	3.5	3.5	10	10
	Number of MTL channels	2	1	2	2	1	2
	Ch 1 localization	HP/HP	HP/HP	HP/HP	HP/HP	ERC/PRC	HP/HP
	Ch 1 IED (% excluded)	4	2	5	4	3	10
	Ch 2 localization	HP/HP	Extra-MTL	HP/HP	HP/PRC	Extra-MTL	HP/HP
	Ch 2 IED (% excluded)	4	4	5	4	3	6
**Electrode 2**	*Hemisphere*	right	right	right	Left	right	right
	Contact Spacing (mm)	10	10	3.5	10	10	3.5
	Number of MTL channels	1	1	2	2	1	2
	Ch 1 localization	HP/PRC	HP/HP	HP/HP	HP/HP	ERC/PRC	ERC/ERC
	Ch 1 IED (% excluded)	3	4	5	2	3	4
	Ch 2 localization	Extra-MTL	Extra-MTL	HP/HP	HP/HP	Extra-MTL	PRC/PRC
	Ch 2 IED (% excluded)	7	2	5	2	3	7

Supplementary Table 1: Participant demographics, experimental task info, and localizations of electrodes. The number of retrieval blocks and trials for the six participants (P1-6) who completed the ambulatory spatial navigation task in the stone and wooden contexts. Localizations of electrode contact pairs for each bipolar recording channel (Ch): hippocampus (HP), perirhinal cortex (PRC), entorhinal cortex (ERC). Extra-MTL indicates contacts that were localized to regions outside of the MTL. Also shown is the percent of data that contained an inter-epileptic discharge (IED) and thus was excluded (% excluded).
