# Supplementary material for: Dynamic neural representations of memory and space during human ambulatory navigation
Source: Nat Commun. 2023 Oct 20;14:6643. doi: 10.1038/s41467-023-42231-4 (PMC10589239; doi:10.1038/s41467-023-42231-4)
Supplement: Supplementary file 3 — Description of Additional Supplementary Files [file 41467_2023_42231_MOESM3_ESM.pdf]

Description of Additional Supplementary Files

Dynamic neural representations of memory and space during human ambulatory navigation

Maoz et al

File Name: Supplementary Movie 1
Description: Visualization of the encoding phase of the ambulatory virtual reality spatial navigation task. (Top left) An illustrative view through the virtual reality headset, synchronized with the (Bottom left) corresponding view of the participant engaging in the task. (Right) The synchronized positional data of the participant is represented by the white line, while the positions of the target halos are indicated by colored circles. Shown is an experimenter performing the task for illustrative purposes (consent to publish obtained).

File Name: Supplementary Movie 2
Description: Visualization of the retrieval phase of the ambulatory virtual reality spatial navigation task. (Top left) An illustrative view through the virtual reality headset, synchronized with the (Bottom left) corresponding view of the participant engaging in the task. (Right) The synchronized positional data of the participant is represented by the white line, with the locations of button presses marked by gray “x”s and the positions of the target halos represented by colored circles. Shown is an experimenter performing the task for illustrative purposes (consent to publish obtained).

File Name: Supplementary Movie 3
Description: Synchronized intracranial EEG (iEEG) activity, movement, and behavior during the ambulatory virtual reality spatial navigation task. (Top left) A camera view provides a real-time demonstration of a participant actively engaged in the task, while (Bottom) synchronized iEEG activity was recorded. (Top middle) The participant’s synchronized positional data is depicted as a white line, with button press locations denoted by gray “x”s and the positions of the target halos indicated by colored circles. (Top right) Normalized instantaneous theta (6-8 Hz) band power (sampling rate of 250 Hz) from an example medial temporal lobe channel, originating from the same participant. Shown is a participant performing the task for illustrative purposes (consent to publish obtained).
