# Supplementary material for: Dynamic neural representations of memory and space during human ambulatory navigation
Source: Nat Commun. 2023 Oct 20;14:6643. doi: 10.1038/s41467-023-42231-4 (PMC10589239; doi:10.1038/s41467-023-42231-4)
Supplement: Supplementary file 7 — Reporting Summary [file 41467_2023_42231_MOESM7_ESM.pdf]

nature portfolio

Corresponding author(s): Dr. Nanthia Suthana

Last updated by author(s): September 13, 2023

Reporting Summary

Nature Portfolio wishes to improve the reproducibility of the work that we publish. This form provides structure for consistency and transparency in reporting. For further information on Nature Portfolio policies, see our Editorial Policies and the Editorial Policy Checklist.

Statistics

For all statistical analyses, confirm that the following items are present in the figure legend, table legend, main text, or Methods section.

n/a	Confirmed	
☐	☒	The exact sample size (*n*) for each experimental group/condition, given as a discrete number and unit of measurement
☐	☒	A statement on whether measurements were taken from distinct samples or whether the same sample was measured repeatedly
☐	☒	The statistical test(s) used AND whether they are one- or two-sided<br>*Only common tests should be described solely by name; describe more complex techniques in the Methods section.*
☐	☒	A description of all covariates tested
☐	☒	A description of any assumptions or corrections, such as tests of normality and adjustment for multiple comparisons
☐	☒	A full description of the statistical parameters including central tendency (e.g. means) or other basic estimates (e.g. regression coefficient) AND variation (e.g. standard deviation) or associated estimates of uncertainty (e.g. confidence intervals)
☐	☒	For null hypothesis testing, the test statistic (e.g. *F*, *t*, *r*) with confidence intervals, effect sizes, degrees of freedom and *P* value noted<br>*Give P values as exact values whenever suitable.*
☒	☐	For Bayesian analysis, information on the choice of priors and Markov chain Monte Carlo settings
☒	☐	For hierarchical and complex designs, identification of the appropriate level for tests and full reporting of outcomes
☐	☒	Estimates of effect sizes (e.g. Cohen's *d*, Pearson's *r*), indicating how they were calculated

Our web collection on statistics for biologists contains articles on many of the points above.

Software and code

Policy information about availability of computer code

Data collection	Neuropace RNS System; Unity game engine (version 2018.4.12f1); OptiTrack MOTIVE (version 2.2.0)
Data analysis	MATLAB 2020a (The MathWorks, Natick, MA, USA); Curve Fitting Toolbox for MATLAB 2020a; Statistics and Machine Learning Toolbox for MATLAB 2020a; Optimization Toolbox for MATLAB 2020a; BOSC toolbox for MATLAB (eBOSC; version June 05, 2019)

For manuscripts utilizing custom algorithms or software that are central to the research but not yet described in published literature, software must be made available to editors and reviewers. We strongly encourage code deposition in a community repository (e.g. GitHub). See the Nature Portfolio guidelines for submitting code & software for further information.

Data

Policy information about availability of data

All manuscripts must include a data availability statement. This statement should provide the following information, where applicable:

- Accession codes, unique identifiers, or web links for publicly available datasets
- A description of any restrictions on data availability
- For clinical datasets or third party data, please ensure that the statement adheres to our policy

The data that support the findings of this study are available from the corresponding authors upon reasonable request.

Human research participants

Policy information about studies involving human research participants and Sex and Gender in Research.

Reporting on sex and gender	Four female and two male participants participated in this study. Recruitment of participants from both male and female sex was part of the study design. Sex- and gender- based analyses were not performed given the small cohort size after subdividing by sex and gender. Additionally, sex- and gender-based analyses were not pursued given that there is was no a priori hypothesis of a difference in episodic memory neurophysiology across sex or gender. As such, results apply to both male and female sex.
Population characteristics	Six participants (33-54 years old) with pharmacoresistant epilepsy participated in the study. These participants were previously implanted with the FDA-approved NeuroPace responsive neurostimulator system (RNS System) for the treatment of their epilepsy. The selection of electrode placements for this procedure (which was performed for all participants before recruitment to this study) were determined by the clinical team based off of treatment criteria. More details can be found in Extended Data Table 1 of the manuscript.
Recruitment	Participants were recruited via phone or email from a database of the University of California Los Angeles. As with any volunteer-based research study, there is a potential for introduction of a self-selection bias that is unavoidable. The recruitment process involved providing potential research participants with a complete overview of the experimental procedure, including the use of virtual reality while freely ambulating while completing an object-place learning paradigm. It is possible that individuals who felt more eager comfortable with navigation and spatial memory were more likely to participate in our study and this could introduce a potential bias impacting behavioral performance. However, it is worth noting that memory performance was highly varied across the six participants (more details in Figure 2) in the study and the effect of this potential bias is likely minimal.
Ethics oversight	All participants volunteered for the study by providing informed consent according to a protocol approved by the UCLA Medical Institutional Review Board (IRB).

Note that full information on the approval of the study protocol must also be provided in the manuscript.

Field-specific reporting

Please select the one below that is the best fit for your research. If you are not sure, read the appropriate sections before making your selection.

☒ Life sciences ☐ Behavioural & social sciences ☐ Ecological, evolutionary & environmental sciences

For a reference copy of the document with all sections, see nature.com/documents/nr-reporting-summary-flat.pdf

Life sciences study design

All studies must disclose on these points even when the disclosure is negative.

Sample size	Six participants (33-54 years old) with pharmacoresistant epilepsy participated in the study. Same size was determined in accordance with previous studies that reported similar effects in freely-moving participants with chronically-implanted NeuroPace RNS Systems (Aghajan et al., 2017, Current Biology; Stangl et al., 2021, Nature); the sample size selected in this study is similar (and greater than) in these prior studies. No statistical methods were used to pre-determine sample size. This sample size was chosen, and the experimental procedured performed individually for each participant, to enable data analyses not only on the group-level (across all recording channels from all participants), but also independently for each participant, in order to investigate the consistency of effects and their reliability across different participants.
Data exclusions	The data from all participants was used for data analyses. Each participant had 4 recording channels. Across all participants, a total of 19 channels were located in MTL regions including the hippocampus, entorhinal cortex, and perirhinal cortex. Recording channels outside of the MTL were excluded from main analyses but evaluated as a control comparison for main effects.
Replication	The experimental procedure was repeated six times independently with six different participants. All methods used to perform this study and analyses needed to replicate the presented findings are detailed in the the Methods section of the manuscript. Data were analyzed on the group-level (across all recording channels from all participants), and across individual participants (averaging across a participant's individual recording channels). Main effects were successfully replicated in all individual participants, as reported in the manuscript.
Randomization	All participants were tested with the same experimental protocol with no separate experimental groups. The task was comprised of learning object positions in two separate environments. The starting environment during encoding for each participant was counterbalanced.
Blinding	We have tested a rare group of participants with pharmacoresistant epilepsy, who have been previously implanted with the NeuroPace RNS System for the treatment of their epilepsy. As such, all experiments were aware of this and not blinded with regards to the participants' condition.

Reporting for specific materials, systems and methods

We require information from authors about some types of materials, experimental systems and methods used in many studies. Here, indicate whether each material, system or method listed is relevant to your study. If you are not sure if a list item applies to your research, read the appropriate section before selecting a response.

Materials & experimental systems

n/a	Involved in the study
☒	☐ Antibodies
☒	☐ Eukaryotic cell lines
☒	☐ Palaeontology and archaeology
☒	☐ Animals and other organisms
☒	☐ Clinical data
☒	☐ Dual use research of concern

Methods

n/a	Involved in the study
☒	☐ ChIP-seq
☒	☐ Flow cytometry
☐	☒ MRI-based neuroimaging

Magnetic resonance imaging

Experimental design

Design type	MRI was used only to determine the localization of electrode contacts within the brain.
Design specifications	MRI was used only for electrode contact localization; thus, participants did not perform an experimental task during MRI scanning.
Behavioral performance measures	No behavioral performance measures were acquired or derived, since participants did not perform an experimental task during MRI scanning.

Acquisition

Imaging type(s)	structural
Field strength	3 Tesla
Sequence & imaging parameters	standard T1- and T2-weighted sequences
Area of acquisition	whole-brain

Diffusion MRI ☐ Used ☒ Not used

Preprocessing

Preprocessing software	MRI data were preprocessed using FSL (FMRIB Software Library, Oxford University, UK; v5.0.11) for image registration with the FLIRT function (default parameters), and ITK-SNAP (version 3.8.0) for visualization and manual segmentation of electrode contacts.
Normalization	MRI images were not normalized.
Normalization template	MRI images were not normalized.
Noise and artifact removal	No noise or artifact removal procedures were applied.
Volume censoring	Volume censoring was not applied.

Statistical modeling & inference

Model type and settings	No model-based analyses were performed using MRI data.
Effect(s) tested	MRI was used only for electrode contact localization; thus, no task- or stimulus-related analyses were performed using MRI data.

Specify type of analysis: ☒ Whole brain ☐ ROI-based ☐ Both

Statistic type for inference (See Eklund et al. 2016)	No statistical analyses were performed using MRI data.

Correction	No statistical analyses were performed using MRI data; thus, no correction methods were applied.

Models & analysis

n/a	Involved in the study
☒	☐ Functional and/or effective connectivity
☒	☐ Graph analysis
☒	☐ Multivariate modeling or predictive analysis
